# Perforated flexible catheters improve joint fluid aspiration in shoulder cadavers

**DOI:** 10.1038/s41598-021-01613-8

**Published:** 2021-11-11

**Authors:** Andreas Hecker, Manuel Waltenspül, Lukas Ernstbrunner, Reto Sutter, Karl Wieser, Samy Bouaicha

**Affiliations:** 1 Department of Orthopedics, Balgrist University Hospital, University of Zurich, Zurich, Switzerland; 2 Department of Orthopaedic Surgery and Traumatology, Inselspital, Bern University Hospital, University of Bern, Bern, Switzerland; 3 Radiology, Balgrist University Hospital, University of Zurich, Zurich, Switzerland

**Keywords:** Medical research, Preclinical research

## Abstract

A fluoroscopically controlled anterior approach in supine position is often used for arthrocentesis of the shoulder, but can lead to a high rate of dry aspirations. The aim of this study was to compare the aspiration performance of rigid needles and flexible catheters used with this approach. We hypothesized that a flexible catheter can significantly improve the amount of the obtained fluid. The glenohumeral joint of ten human cadaveric shoulder specimens were sequentially filled with 5, 10, 20 and 30 mL of contrast agent. For each volume the maximum aspirated amount of contrast agent with 4 different aspiration devices (20 gauge needle, 16 gauge needle, 16 gauge flexible catheter and 16 gauge perforated flexible catheter) were compared. All aspirations were done in supine cadaver position from anterior under fluoroscopic control. The aspirated amount of fluid was significantly higher using the 16 gauge perforated flexible catheter (p = 0.002–0.028) compared with all other devices when 5, 10 and 20 mL of contrast agent were in the joint. This perforated flexible catheter aspirated 80–96% of the available fluid while the standard 20 gauge needle aspirated 40–60%. Using a 16 gauge perforated flexible catheter in a supine anterior arthrocentesis technique results in aspiration of most of the fluid in human cadaveric shoulder specimens, while standard needles aspirate only about 50% of it. This can be clinically relevant when there is very little synovial fluid available and might reduce the number of insufficient aspirations.

## Introduction

Arthrocentesis of the hip and knee joint is well studied and leads to sufficient sensitivity and specificity to guide further treatment^1^. This is especially important in revision surgery because treatment varies when infection is present and knowing the causative pathogen is critical. The surgeon might rather choose a two-stage procedure if a multi-resistant germ is present or is able to directly administer the correct antibiotic treatment in a one-stage procedure^2^. For infection workup in shoulder surgery the literature is less clear. A recent study reports a low sensitivity of shoulder aspirations of only 33%^3^ while other studies provide a large range for the sensitivity of standard joint aspirations between 16.7^4^ and 81.3%^2^. Another problem is the rate of dry aspirations reported as high as 43%^3^. Irrigation of the shoulder joint in case of dry aspiration does not improve sensitivity like in hip and knee aspirations^3,5^. Nevertheless, arthrocentesis is recommended to assess for infection prior to revision shoulder surgery^6^.

Shoulder joint aspiration for fluid collection can be done from anterior or posterior in sitting, beach-chair, supine or lateral decubitus position^7^. Due to gravity the intra-articular fluid is localized in the inferior recess in sitting and beach-chair position and in the posterior recess in supine position. If external rotation is applied even in a sitting patient the fluid can be found in the posterior recess^8^. In our institution shoulder joint aspirations are performed in supine position with anterior–posterior fluoroscopic projection, to confirm the correct location of the needle. The other positions described above can potentially lead to difficulties to perform the procedure if the patient collapses and the adjustment of the fluoroscopic field-of-view is more time consuming. However, the supine position might be not ideal to achieve the main goal of fluid aspiration if not much fluid is present because due to gravity the fluid is located in the posterior recess away from the anteriorly introduced needle.

The aim of this study was to compare the performance of different aspiration devices used in an anterior approach. We hypothesize that by using a flexible plastic catheter for shoulder joint aspirations instead of a rigid needle the amount of the obtained fluid can be significantly improved.

## Materials and methods

Eleven human cadaveric shoulder joint specimens including the proximal humerus, scapula and clavicle with intact capsule, tendons, muscles and skin were used for this investigation. Cadavers with previous surgery, osteoarthritis, rotator cuff tears or any other change of normal anatomy, were excluded from this study to represent results in a functional shoulder joint with normal anatomy. The cadavers were obtained through a donor platform (Science Care, Phoenix, Arizona, USA). The platform guarantees that an informed consent of the next of kin or an authorized representative was obtained. The study was approved by the local ethics committee and handling of the subjects was performed in accordance with the guidelines of the Declaration of Helsinki and the Swiss human research act. The first cadaveric shoulder was prepared to establish the technique. In this pilot shoulder a dry arthroscopy was performed using the Neviaser portal and an anterolateral portal to confirm and observe the intra-articular position and course of the inserted needles and catheters.

The ten remaining shoulders were then treated according to the following protocol.

The position of the shoulder joints was supine with the arm adducted and with slight external rotation to tension the anterior capsule. First a 16 gauge catheter was brought into the gleno-humeral articulation through an antero-inferior approach, medial and inferior to the coracoid process. The described position was chosen to avoid impairment of the later aspirations. This catheter was exclusively used for the instillation of the iodine contrast agent. To confirm the intra-articular position of the catheter, 1 mL of iodine contrast agent (Iopamiro 200, 200 mg/mL of iodine; Bracco, Milan, Italy) was injected and an antero-posterior radiograph was obtained.

**Figure 1:**
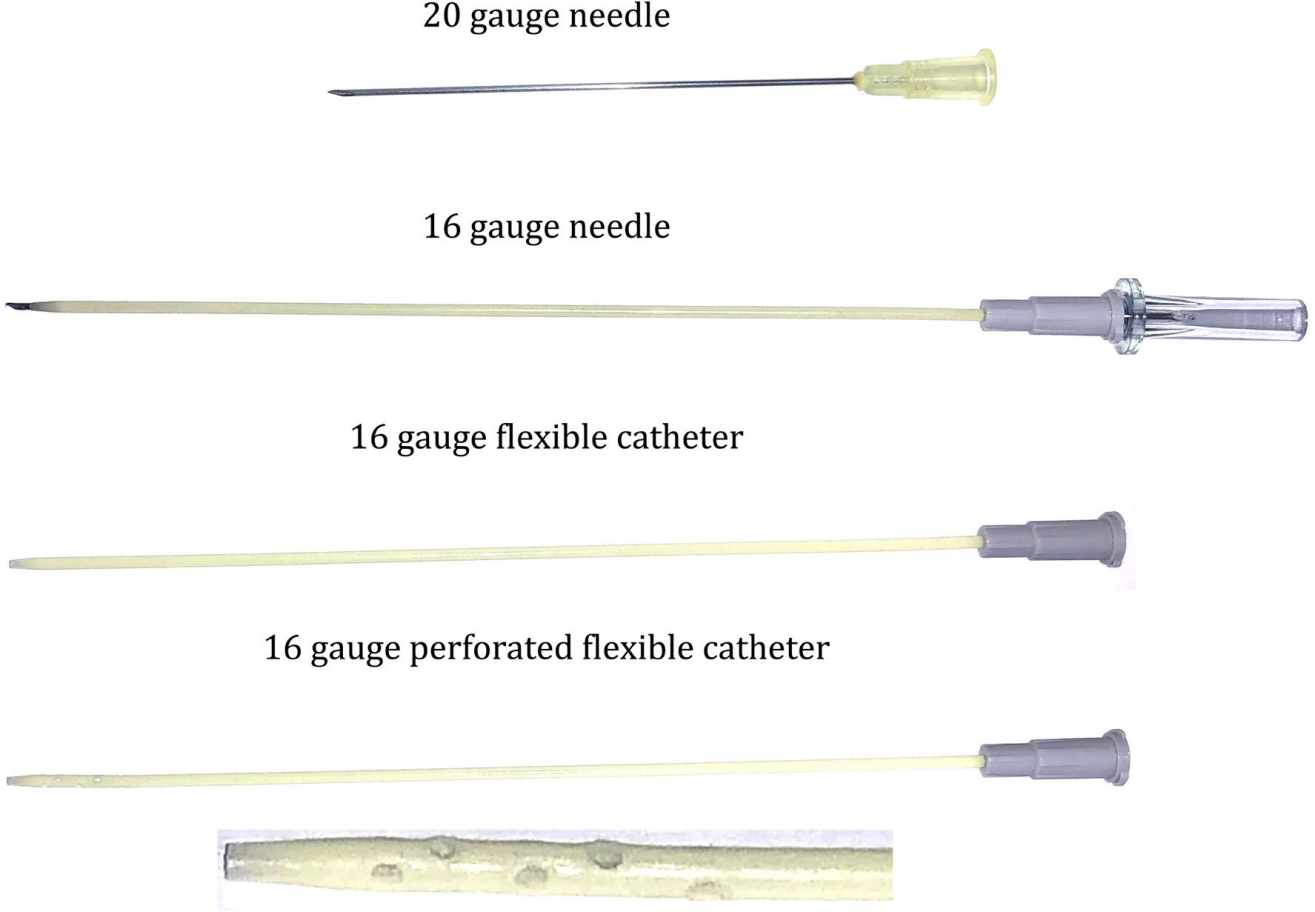
Needles/catheters used in the experiment. The tip of the perforated catheter is displayed zoomed for better visualization of the perforations.

Then the shoulder aspiration was performed through an anterior approach under fluoroscopic control after injection of different amounts of contrast agent. The entry point was chosen directly lateral to the tip of the coracoid process aiming slightly superior and medial to reach the superior part of the articulation. The same entry point was used for all aspirations. The following needles and techniques were applied in a sequential fashion: A 20 gauge needle [20GN], (0.9 × 70 mm, Braun Melsungen AG, Germany), a 16 gauge needle [16GN], (1.7 × 133 mm, BD Medical, Australia), a 16 gauge flexible catheter [16GFC], (1.7 × 133 mm, BD Medical, Australia) and a 16 gauge perforated flexible catheter which was modified at the tip [16GPFC], (1.7 × 133 mm, BD Medical, Australia). The modified catheter was made by drilling ten holes of 1.7 mm around the end of the catheter starting 5 mm from the tip. The first six holes were drilled through both sides of the catheter 5 mm apart from each other. Then four additional holes were applied perpendicular and in-between the first ones. The used needles and catheters are displayed in Fig. 1.

After insertion of a needle or catheter the aspiration of the previously instilled contrast agent was attempted and the needle subsequently slowly pulled out of the joint under permanent negative pressure. Then the aspirated amount of fluid was documented and the aspirate was injected back into the shoulder joint. For the plastic catheters a 1 mm flexible metal guide wire was inserted through the catheter after removal of the rigid insertion needle and the intra-articular position was confirmed under fluoroscopy. After all four needles/catheters were applied the aspirations were repeated with increased volumes (5 mL, 10 mL, 20 mL and 30 mL) of contrast agent in the joint. The order was always first 20GN, then 16GN, then 16GFC and finally 16GPFC for each volume. Every additional filling was followed by fluoroscopic confirmation of the intra-articular location of the contrast agent. In all cases the insertion depth of the needle or catheter was measured and documented.

**Figure 2:**
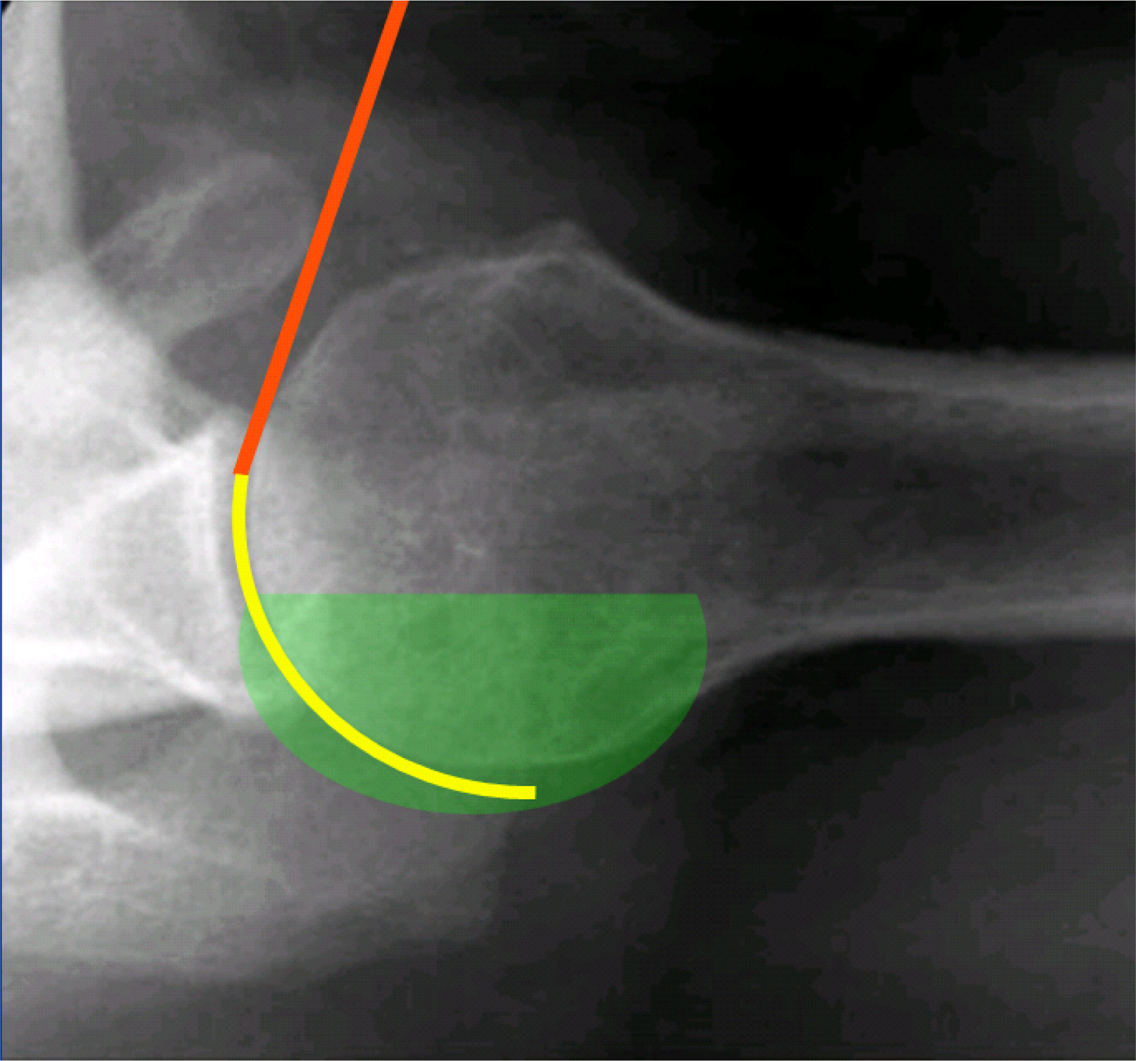
Illustration of the course of a needle and catheter during arthrocentesis. An axial radiograph of a right shoulder in supine position is shown. The abducted position in this radiograph is used for better illustration only. During the aspirations in this study the shoulder was adducted. The green area represents the fluid in the joint, the orange line represents a rigid steel needle and the yellow line represents a flexible catheter which is advanced into the posterior recess.

The procedure using a flexible catheter for shoulder aspiration is performed as follows: The identification of the bony landmarks of the shoulder, especially of the coracoid process for the proper entry point is the same as for rigid needle aspirations. Then an anterior–posterior (a.p.) fluoroscopic view of the gleno-humeral joint is acquired. Further the needle with catheter is inserted through the skin directly lateral to the coracoid process and the needle is advanced aiming to the upper third of the gleno-humeral articulation. After passing the shoulder joint capsule a loss of resistance is felt. The fluoroscopic control should show the needle tip directly lateral to the glenoid’s upper third. The rigid needle is then kept in place, while the flexible plastic catheter is advanced further posterior into the gleno-humeral articulation. No strong resistance should be felt by doing so. The catheter is advanced as far as possible towards the posterior capsule. The rigid needle is removed while the flexible catheter is kept in place. A syringe is attached to the catheter and the plunger is slowly pulled. The catheter is kept in this position as long as joint fluid can be aspirated. If no more fluid is obtained, the catheter is slowly pulled out of the joint while negative pressure is maintained by pulling the plunger. If joint fluid is again streaming into the syringe doing this maneuver, the catheter is kept in place until no more fluid is obtained and then again pulled back a little in the same fashion. After aspiration the catheter is pulled out of the joint and skin and the puncture wound is closed with a sterile dressing. Figure 2 shows schematically the difference between a rigid steel needle and a flexible plastic catheter.

### Statistics

The minimally needed volume of aspirated joint fluid to perform an infectional workup is 2 mL^9^. Therefore, this volume was used as the minimal important difference between the different aspiration techniques with an estimated standard deviation of 1 mL.

An a priori power analysis in form of a paired t-test with a significance level of 0.05 (type I error), mean of the differences of 2 mL with a standard deviation of 1 mL and a chosen power of 0.8 reveal a minimal sample size of 4 shoulders. To account for the only estimated standard deviation and to increase the power we used 10 shoulders in this experiment.

Normal distribution was tested with the Shapiro–Wilk test. Continuous variables are depicted as median and 25% and 75% quartiles. Different aspiration techniques as well as puncture depths were compared using the Friedman’s Two-Way Analysis of Variance. Significance was set as p < 0.05 with use of Bonferroni adjustment.

### Ethics approval

The study was approved by the Local Review Board, Kantonale Ethik Komission (KEK) Zurich, Switzerland, Application BASEC-Nr. 2018-00588.

## Results

Seven male and three female cadaver shoulders of Caucasian race with a mean age of 54 (range 34–63) years were assessed. Each five were left and right shoulders.

In one shoulder a dehiscence of the capsule was detected fluoroscopically after filling with 20 mL and therefore measurements with 20 mL and 30 mL filling could not be carried out.

The rigid needles could be inserted into the joint less than half as deep as the flexible catheters which was significantly different (p < 0.001). The median insertion depth was 49 mm (range 43–59 mm) for the 20GN, 43 mm (range 35–53 mm) for the 16GN, 123 mm (range 108–123 mm) for the 16GFC and 123 mm (range 113–125 mm) for the 16GPFC. No significant difference regarding insertion depth was seen between the two rigid needle techniques (p = 0.414) as well as between the two flexible catheter techniques (p = 1.000).

Table 1 shows median values of the amount of aspirated fluid of each volume from 5 to 30 mL. The 16GPFC showed the highest aspiration capacity with 4.8 of 5 mL, 9.0 of 10 mL, 17.5 of 20 mL and 24.0 of 30 mL of sequential filling quantities. Using the standard 20GN 2.0 mL, 5.0 mL, 12.0 mL and 15.5 mL of fluid could be aspirated respectively. The 16GN showed values of 2.3 m, 3.8 mL, 8.0 mL and 11.0 mL while with the 16GFC 1.5 mL, 1.8 mL, 4.0 mL and 5.0 mL were aspirated.

**Table 1:** Amount of fluid aspirated (mL).

	20 gauge rigid needle Median (Q1, Q3)	16 gauge rigid needle Median (Q1, Q3)	16 gauge flexible catheter Median (Q1, Q3)	16 gauge perforated flexible catheter Median (Q1, Q3)
5 mL filling	2.0 (40%) (1.0, 4.5)	2.3 (46%) (0.0, 4.3)	1.5 (30%) (0.9, 4.3)	4.8 (96%) (4.0–5.0)
10 mL filling	5.0 (50%) (2.4, 9.0)	3.8 (38%) (0.9, 9.0)	1.8 (18%) (0.0, 8.6)	9.0 (90%) (8.5, 9.6)
20 mL filling	12.0 (60%) (6.8, 14.3)	8.0 (40%) (1.5, 16.3)	4.0 (20%) (2.0, 13.3)	17.5 (88%) (15.5, 18.3)
30 mL filling	15.5 (52%) (11.0, 24.0)	11.0 (37%) (0.8, 17.0)	5.0 (17%) (2.0, 20.0)	24.0 (80%) (19.0, 25.8)

n = 10 (5 mL, 10 mL); n = 9 (20 mL, 30 mL); Q1 = 25% quartile, Q3 = 75% quartile.

**Figure 3:**
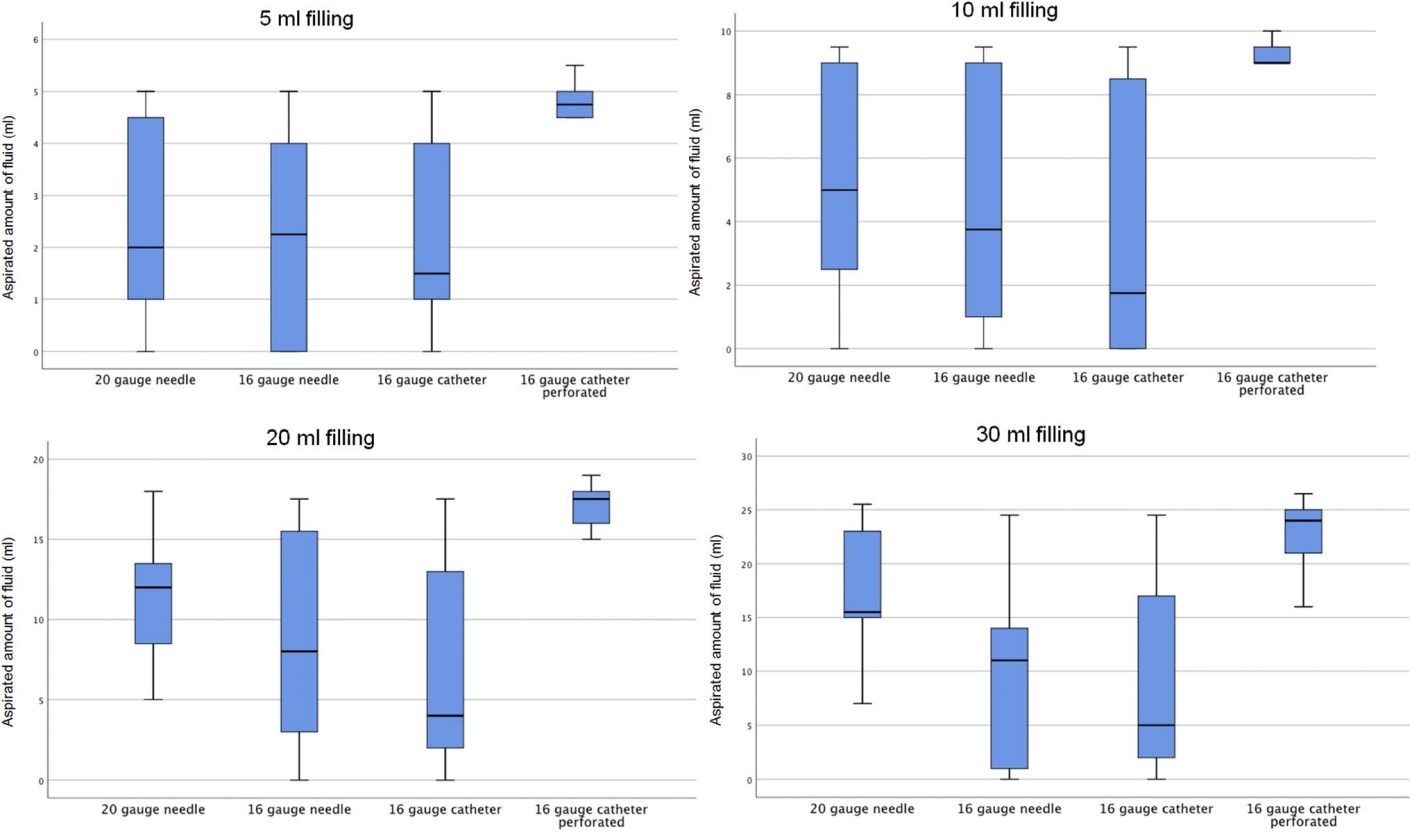
Comparison of aspiration devices. The amount of aspirated fluid is displayed for each needle/catheter for filling amounts of the shoulder joint of 5, 10, 20 and 30 mL. The black bold line represents the median.

The aspirated amount of fluid was significantly higher using the 16GPFC compared with all other aspiration devices when 5, 10 and 20 mL of contrast agent were available in the joint. Regarding 30 mL of filling the perforated catheter was significantly superior to the 16GFC (p = 0.049) and 16GN (p = 0.028). For the standard 20GN statistical significance was not reached for 30 mL of filling despite a considerable difference of the medians of 8.5 mL. The comparison of all techniques regarding each filling amount is displayed in Fig. 3.

## Discussion

This experimental cadaver study shows, that a significantly increased amount of fluid can be obtained with shoulder joint aspiration when a perforated flexible catheter is used compared to standard rigid needles. With the perforated flexible catheter 80–96% of the joint fluid could be aspirated, compared to 40–60% using the standard rigid needle. Such a perforated catheter can be particularly helpful if there is very little fluid in the joint. Without the perforations around the tip the flexible catheter did not show better results than the 16 and 20 gauge rigid needles.

We hypothesized that by using a flexible plastic catheter for shoulder joint aspirations the amount of the obtained fluid can be significantly improved. Regarding the flexible perforated catheter we were able to confirm this hypothesis. Without perforations the catheter did not meet our expectations and led to even lower aspiration volumes than the conventional needle, despite positioning in the posterior recess. Presumably aspiration of synovial tissue that consecutively occludes the single opening of the catheter at the tip may be detrimental to aspirating joint fluid. This might be worse in a flexible catheter in comparison to a rigid needle, because it lies deep in the posterior recess and has inevitable contact to the synovia. We observed this phenomenon during the pilot shoulder setup using arthroscopy and therefore newly developed the above described modified perforated catheter.

Obviously all catheters and needles can lead to a successful aspiration if enough fluid is in the joint. For a complete infectiological workup including the analysis of cell count, crystals and bacteriological culture at least 2 mL of joint fluid is necessary^9^. In our clinical experience, less than 1 mL synovial fluid is obtained in many shoulder aspirations and therefore, a complete analysis is often not possible. Considering the results of this study, a perforated flexible catheter may lead to a sufficient amount of aspirated synovial fluid in these cases.

Attempts to improve the outcome of arthrocentesis are also known for other joints. In the knee joint manual compression improves the success of joint aspirations^10^. Mechanical compression showed a significant higher amount of obtained fluid and lowered the rate of dry aspirations compared to pure manual compression when a circumferential elastomeric compressive brace was applied^11^. Unfortunately this technique is not transferable to the shoulder joint due to anatomic reasons including a large soft tissue covering. Moreover, a circumferential brace would obscure the puncture site. Another study concentrated on the position of the patient while performing knee arthrocentesis and found that more fluid was obtained in supine position compared to sitting position^12^. Regarding shoulder aspirations to our knowledge no comparison between different positions exists. Based on our clinical experience, however, the supine position is the most effective method for performing shoulder aspirations. For this reason, we have tried to develop a technique for this particular posture. Perforated drains are routinely used after surgery to drain deep wounds and joints. To our knowledge no study exists that compares perforated and not perforated drains. The technique introduced in this investigation has never been described for joint aspirations but could potentially reduce dry aspirations respectively increase the amount of obtained fluid and therefore increase sensitivity of arthrocentesis of the shoulder joint. This is a first report of preclinical data regarding a new promising shoulder aspiration technique.

Due to its character our study has limitations. We only used cadavers with native shoulder joints and no relevant arthritis or rotator cuff tears. Aspiration of replaced or worn out joints might lead to different results. The aim of this study was to examine the performance of different aspiration devices under normal anatomical conditions. In case of absence of the rotator cuff or in case of arthritis the aspiration usually becomes easier because the joint space enlarges and/or more joint fluid is present. No dry aspirations occurred, because great value was put on a standardized placement of the needle in the shoulder joint. Which means in some cases the needle location was changed a few times before aspiration until the perfect location was achieved (which is not always possible in a patient). Also common clinical problems during arthrocentesis like pain, muscle contractions and movement of the patient, which can potentially lead to “dry taps” did not exist. Finally, at least 5 mL of fluid was available in the joint, which makes unsuccessful aspiration unlikely. The results shown above were evaluated only in supine position with an anterior approach, other positions and approaches could show different results. We could not assess whether the advancement of the flexible catheter towards the posterior aspects of the shoulder joint would lead to more pain compared to the standard technique with the rigid needle, but a proof of concept in cadavers was a prerequisite before inception of a clinical study. It has to be mentioned, that we used contrast agent in the experiment, which due to different viscosity might behave slightly different than synovial fluid. Another limitation is, even though the aspirate was injected back after every aspiration, a small amount of it might have gone lost in the tissue and therefore would not have been available for next aspiration device. In this case the results of the 16GPFC might be even underestimated, because it was always the last device used for each filling volume. Moreover a perforated flexible catheter like described above is not commercially available at the moment and has to be developed and manufactured before this technique can be implemented in clinical practice. Also the ideal diameter of such a catheter in terms of fluid dynamics is not clear yet and could not be tested in our protocol. It is subject of our future work to develop and implement perforated catheters of different sizes and diameters to evaluate which one is able to maximize the aspiration capacity with arthrocentesis. A randomized clinical trial will be necessary to evaluate the feasibility of this new technique in patients and to assess its clinical efficacy.

In conclusion using a 16 gauge perforated flexible catheter in a supine anterior arthrocentesis technique results in aspiration of most of the fluid in human cadaveric shoulder specimens, while standard needles aspirate only about 50% of it. This can be clinically relevant when there is very little synovial fluid in the joint, and may increase the sensitivity of shoulder joint aspirations by reducing the number of insufficient aspirations.

## Data Availability

The project data is completely available on a save server of University Hospital Balgrist.
